# A META ANALYSIS ON THE ASSOCIATION OF NOVEL PLASMA BIOMARKERS TESTING FOR EARLY DETECTION OF ALZHEIMER’S DISEASE

**DOI:** 10.1093/geroni/igad104.3142

**Published:** 2023-12-21

**Authors:** Charles Dunnam

**Affiliations:** Wichita State University, Wichita, Kansas, United States

## Abstract

The latest research confirms the heterogeneity of Alzheimer’s Disease Related Dementia (ADRD). This justifies a meta-analysis study on the association of novel plasma biomarkers with screening assessments for early detection of ADRD. The conceptual framework model will be analyzed from the 2020 edition of the GSA KAER Toolkit with updates. The purpose of the current study is two-fold. First, to determine the need of a universal protocol method for early assessment of ADRD by primary care teams. Second, implementation of Food and Drug Administration (FDA) approved novel plasma biomarkers testing as a follow-up protocol to a screening assessment. The two suggested protocols have recently established Medicare coverage. The study will conclude with an extensive review of the known novel plasma biomarkers that are available for testing, at present and a brief overview of available screening assessments for early detection of ADRD.

